# Clinician Beware, Giant Ovarian Cysts are Elusive and Rare

**DOI:** 10.7759/cureus.6753

**Published:** 2020-01-23

**Authors:** Christine E Albers, Eukesh Ranjit, Amit Sapra, Priyanka Bhandari, Waiz Wasey

**Affiliations:** 1 Family and Community Medicine, Southern Illinois University, Springfield, USA; 2 Family Medicine, Southern Illinois University School of Medicine, Springfield, USA

**Keywords:** ovarian mass, serous cystadenoma, giant ovarian tumors, pelvic mass, constipation, abdominal discomfort, rare presentations

## Abstract

Giant ovarian cysts, which are described in the literature as measuring more than 10 cms in size in their largest diameter, are rare in occurrence. With the availability of multiple imaging modalities and routine physical examinations, it has become even rarer to find such cases. Ovarian serous cystadenomas, which are benign tumors arising from the ovarian epithelium, represent the most common type.

We present a case of a 58-year-old female who came to establish primary care in our clinic. She reported ongoing symptoms of constipation, abdominal discomfort, bloating, as well as intermittent postmenopausal bleeding for the past few months. The patient reported taking over-the-counter medications for her predominant gastrointestinal symptoms with no improvement at all.

Transvaginal ultrasonography (TVUS) and magnetic resonance imaging (MRI) of the pelvis revealed the presence of giant bilateral ovarian masses measuring more than 17 X 10cms each. Further testing revealed highly elevated levels of tumor markers cancer antigen 125 (CA-125) and human epididymis protein 4 (HE-4). The patient subsequently underwent total abdominal hysterectomy (TAH) and bilateral salpingo-oophorectomy (BSO). Her histopathology report revealed the presence of bilateral benign cystadenomas.

From a primary care physician's perspective, this case highlights the importance of possible rare pathologies that can present with symptoms of a completely unrelated organ system. Even with the rarity of these cases, a clinician may encounter such a case in their everyday practice. Patients can endorse a plethora of vague complaints, often masquerading other entities seen commonly in the clinic.

## Introduction

An ovarian cyst is a common condition seen in clinical practice and is a common cause of pelvic/adnexal masses [1]. Giant ovarian cysts, on the other hand, are rare findings in current clinical practice. Historically, ovarian cysts measuring up to 148.6 kg (328 lb) have been recorded in the medical literature [2].

Advancement in imaging modalities and routine physical examinations have enabled clinicians to diagnose ovarian cysts while they are at a relatively smaller size.

Giant cysts are often misdiagnosed due to their vague symptoms and non-specific findings. Also, a delay in diagnosis, especially among asymptomatic patients, is seen due to their varied presentations.

## Case presentation

Our patient is a 58-year-old Caucasian female with a past medical history of hypertension, hyperlipidemia, class III obesity, diabetes mellitus, tobacco use, obstructive sleep apnea, gastroesophageal reflux disease, and hypothyroidism who presented to the Family Medicine clinic for evaluation of vague abdominal symptoms, such as constipation, abdominal discomfort, bloating, as well as intermittent postmenopausal bleeding, for the past six to eight months.

She denied being sexually active for the last five years. She did not endorse vaginal discharge, fever, chills, abdominal pain, flank pain, dysuria, hematuria, pelvic discomfort, low back pain, or noncompliance with thyroid medication. Gynecologic history was significant for menarche at age 12 and premature menopause at age 38, and surgical history was significant for having undergone a cesarean section at 30 years of age. Family history was significant for lymphoma in mother, breast cancer in paternal aunt, and pancreatic cancer in maternal grandmother.

During her office visit, her pelvic exam revealed a normal bimanual exam without masses, fullness, or cervical motion tenderness. The uterus was normal by palpation. Papanicolaou smear was also done at this visit. Her cervical cytology was negative for any intraepithelial lesion as well and negative for high-risk HPV.

Lab work, including thyroid-stimulating hormone (TSH), complete blood count (CBC), comprehensive metabolic panel (CMP), follicle-stimulating hormone (FSH), and luteinizing hormone (LH) were unremarkable. Her bilateral mammogram was reported as being benign.

Transvaginal ultrasound was significant for bilateral multilocular cystic masses with thin septations and hypoechoic fluid suspicious for serous or mucinous cystadenoma and thickened endometrium of 17 mm.

**Figure 1:**
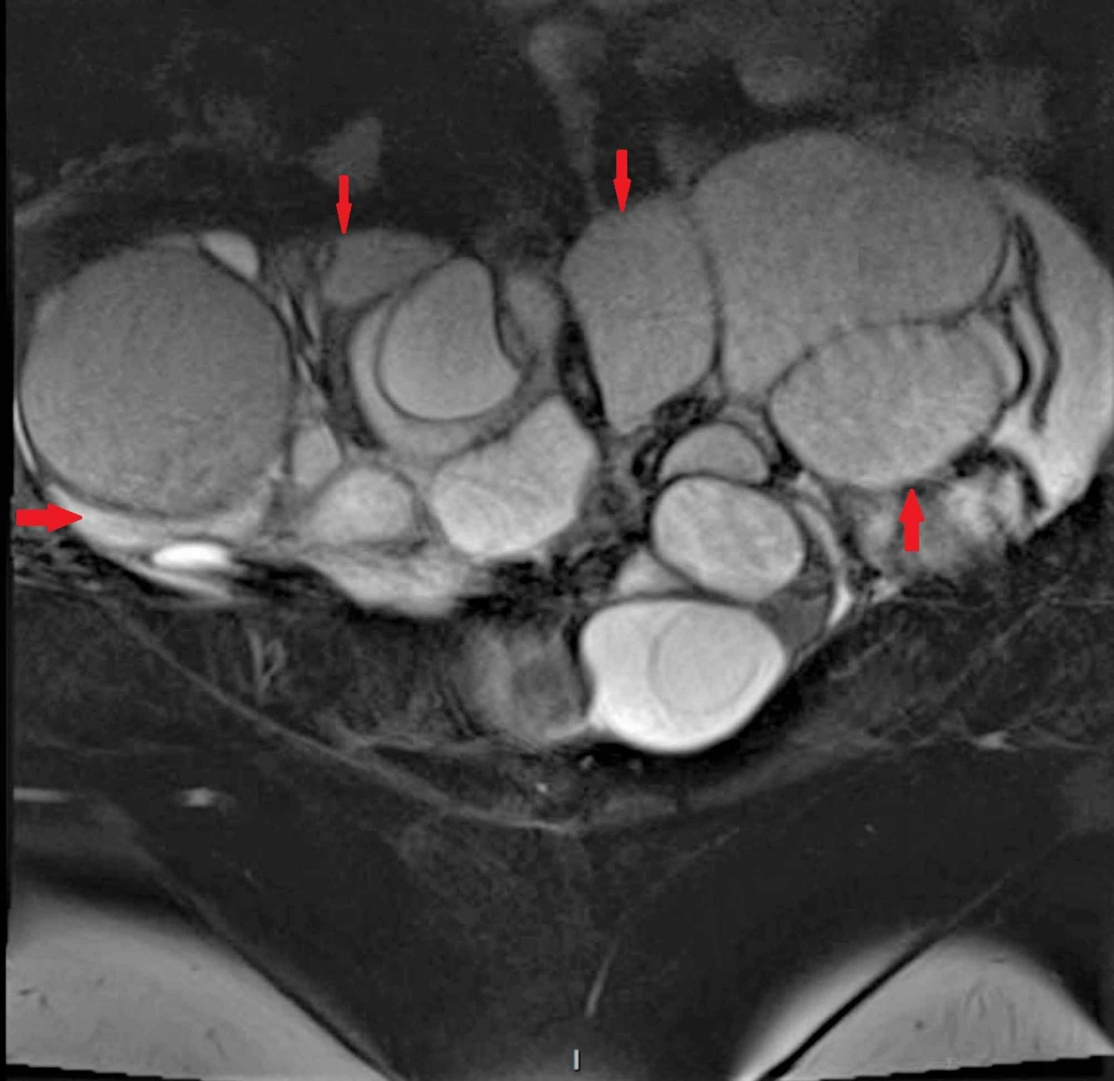
Coronal section of the patient's pelvic MRI showing severely enlarged bilateral ovarian masses with numerous cystic spaces separated by thin septations

**Figure 2:**
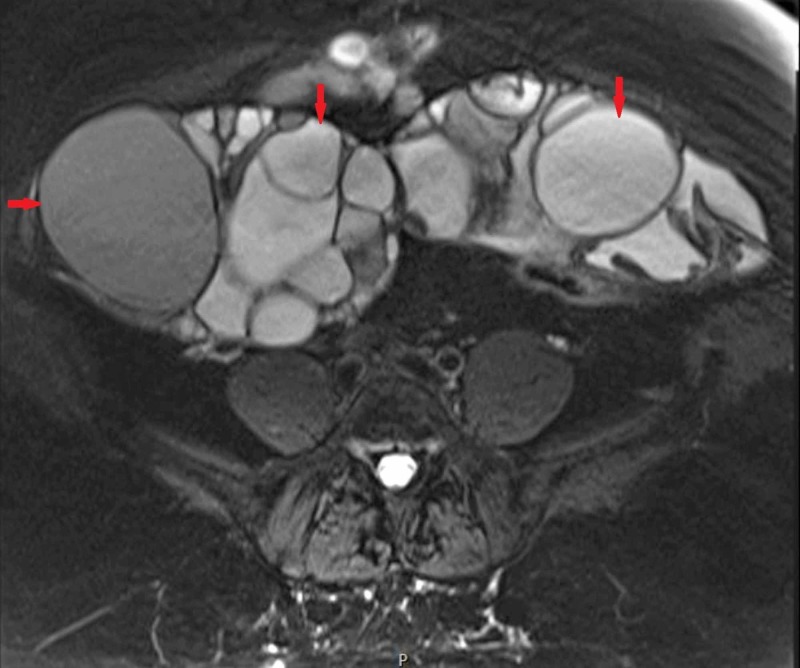
Transverse section of the patient's pelvic MRI showing severely enlarged bilateral ovarian masses with numerous cystic spaces separated by thin septations

Magnetic resonance imaging (MRI) without contrast of the pelvis confirmed bilateral multicystic ovarian masses measuring 16 x 10 cm and 17 x 9 cm with the presence of thick septations and papillary-like projections (Figures 1-2). The patient was referred to the outpatient obstetrics and gynecology clinic for further management, where she underwent an endometrial biopsy that revealed benign findings.

**Figure 3:**
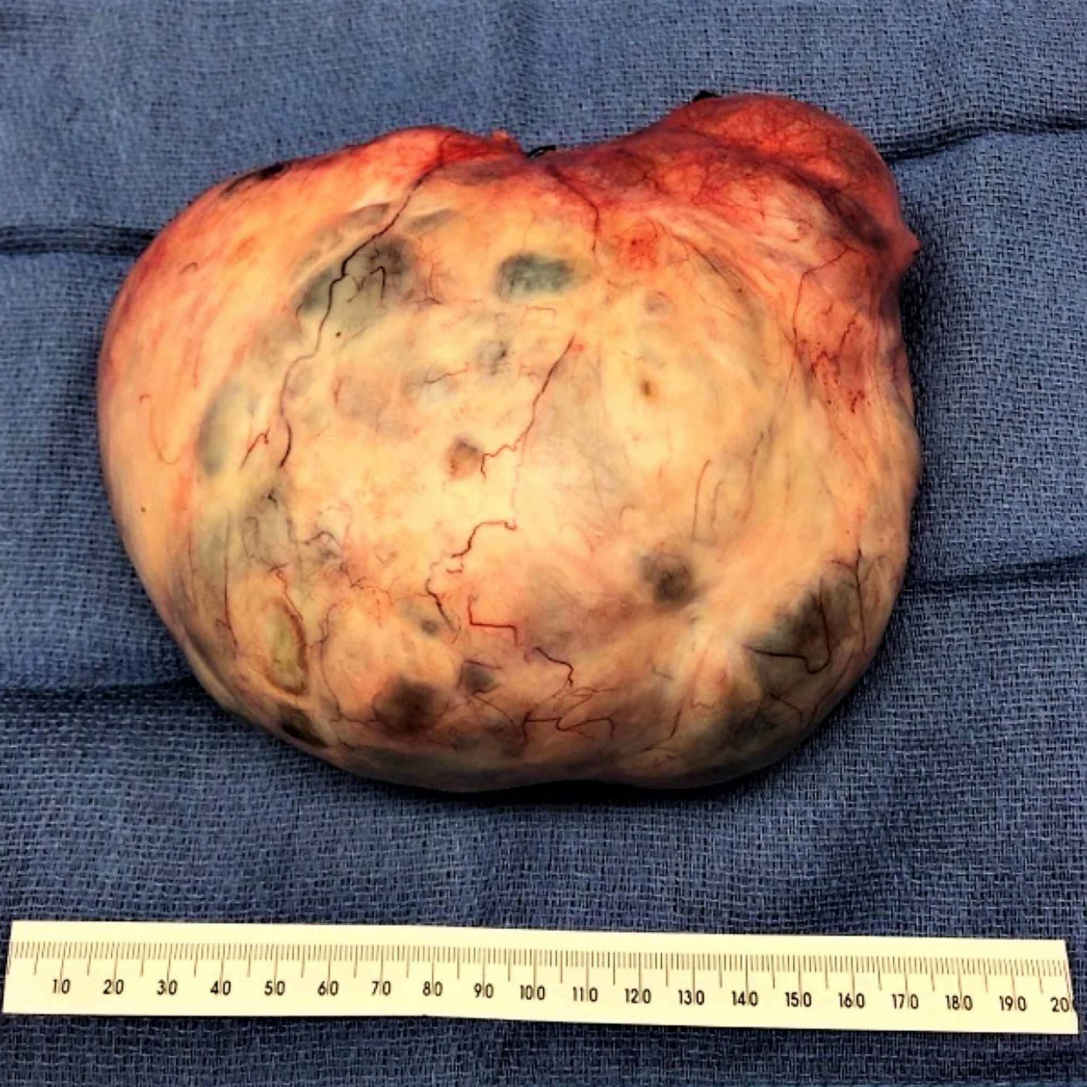
Postoperative specimen of the left ovarian mass measuring 18 cm X 14 cm X 8 cm; the surface is intact and has smooth contours

**Figure 4:**
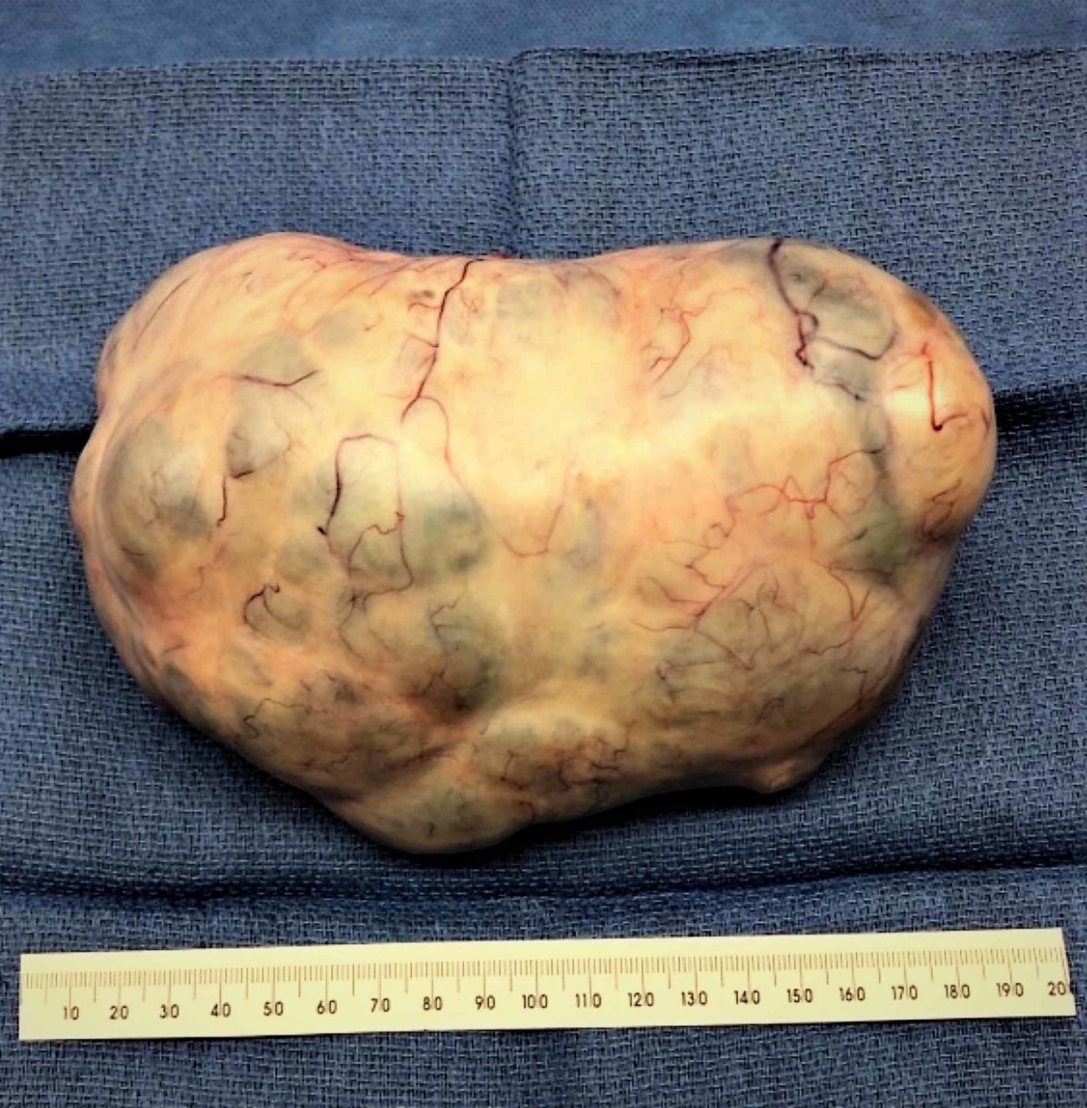
Right ovarian mass, 17 cm X 11 cm X 9 cm in dimension

Cancer antigen-125 (CA-125) and human epididymis protein 4 (HE4) performed at that visit were elevated at 272 U/ml and 229 pmol/L. The patient was then referred to Gynecological Oncology for surgical intervention. She underwent exploratory laparotomy, bilateral salpingo-oophorectomy, and total abdominal hysterectomy (Figures 3-4).

**Figure 5:**
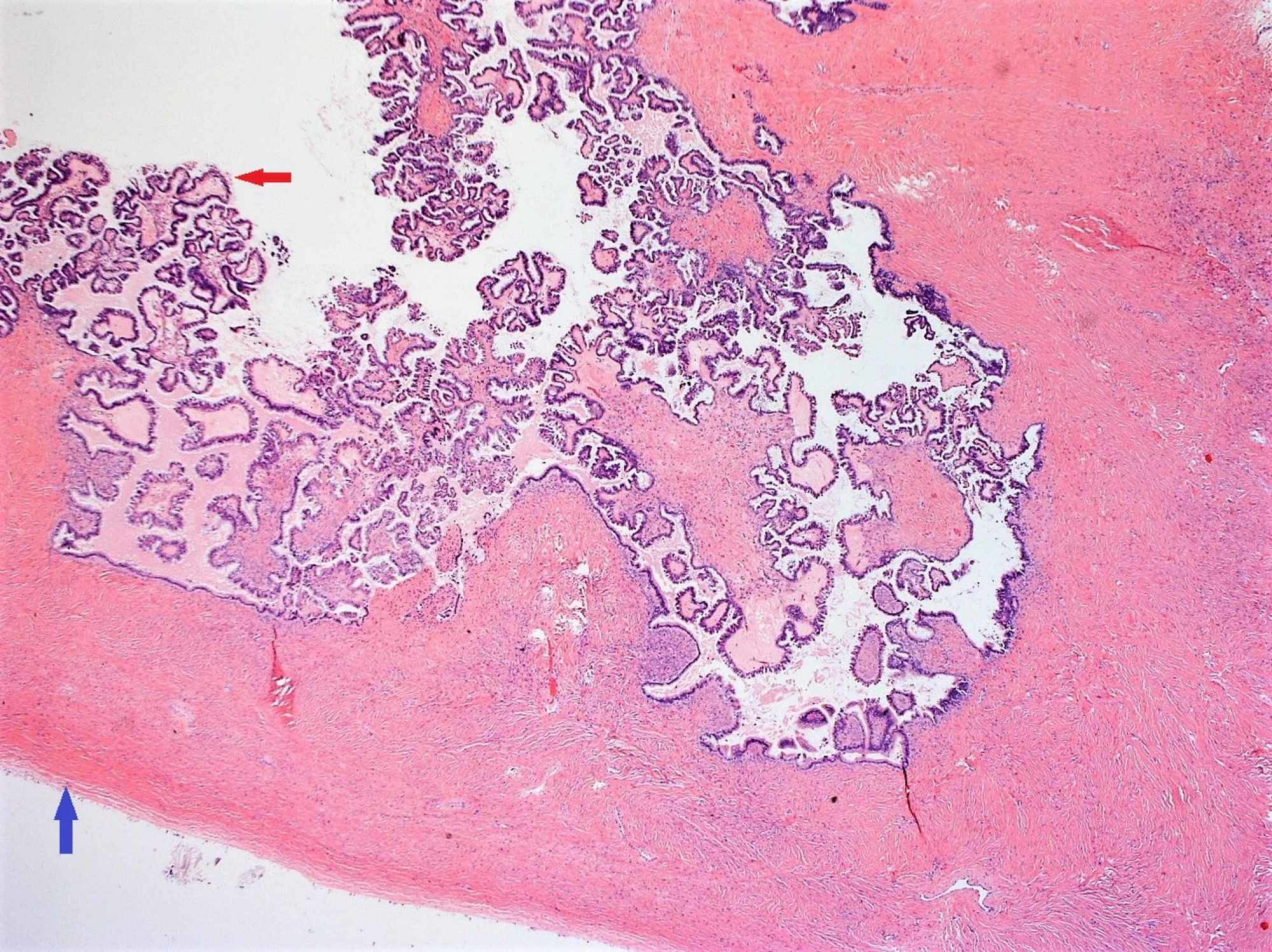
Red arrow: Papillary projections in the inner surface of the cystic tumor; Blue arrow: Smooth outer wall of the cystic tumor

**Figure 6:**
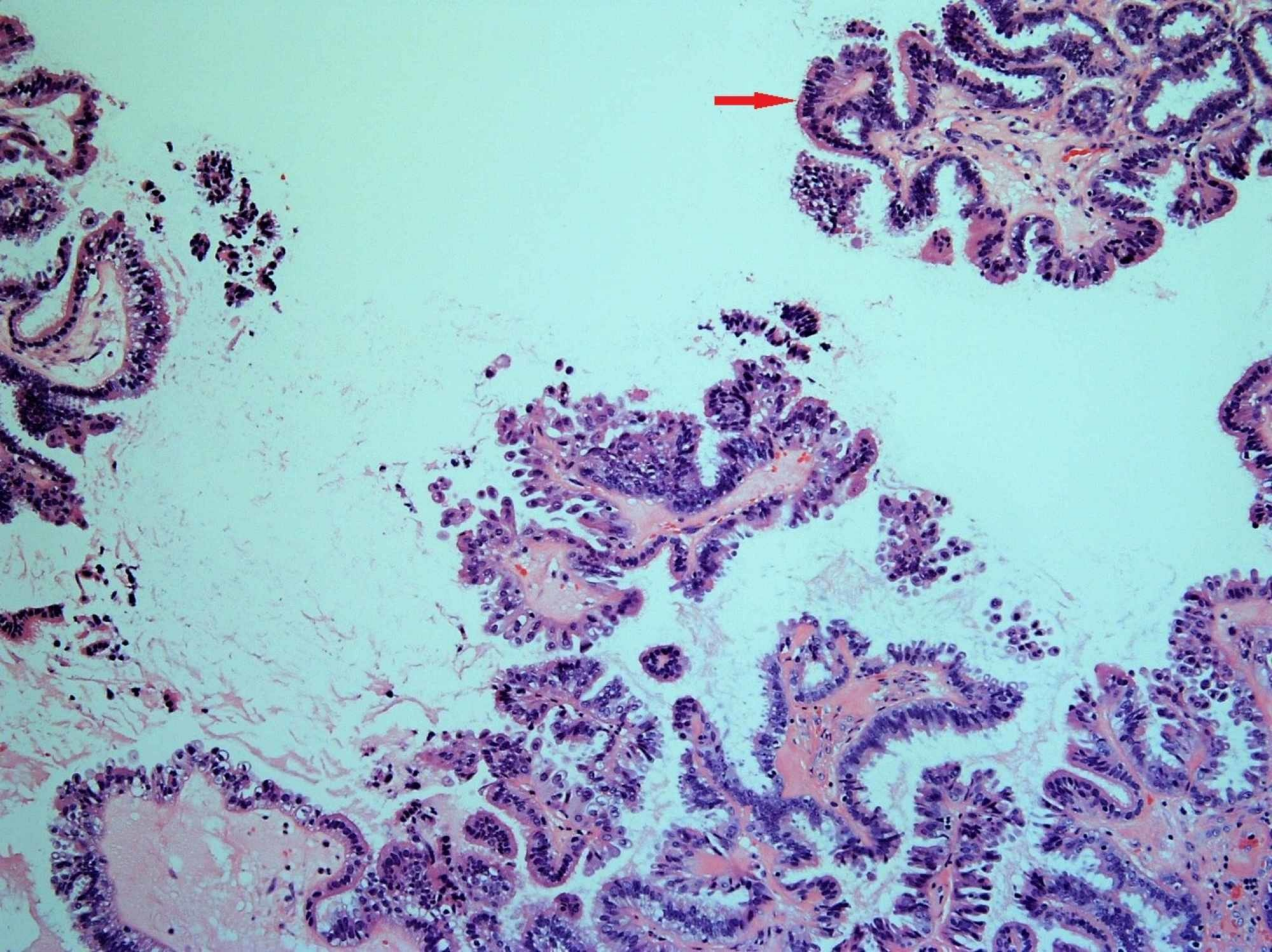
Bluish cells on the inner surface of the cystic tumor, indicating their serous type

The histopathology report revealed bilateral serous cystadenomas with focal epithelial proliferation to the right ovarian mass and endometrial polyps, adenomyosis, and multiple leiomyomas in the uterus (Figures 5-6) No further adjuvant treatment was required. The patient tolerated the procedure well. She has recently had her first postop visit and her surgical incision has healed well.

## Discussion

Epidemiology

Ovarian cysts are a very commonly encountered condition in females [1]. Worldwide, about 7% of women are reported to have an ovarian cyst at any point in their life. Studies have reported the rates of ovarian cyst in postmenopausal women in the United States and Europe to be 18% and about 21%, respectively [3].

Giant ovarian cysts, which are generally described as more than 10 centimeters in size in their largest diameter [4], are rare in occurrence. Due to rarity and a lack of standardized definition, the incidence or prevalence rate of a giant ovarian tumor could not be ascertained. Giant ovarian cysts are seen more frequently between the third and sixth decades of life [5-6]. A review of the literature does show sporadic case reports of these cysts, especially in postmenopausal women [7-11].

Demography

Per the 2014 World Health Organization (WHO) classification and tumor morphology, primary ovarian tumors are categorized into three types of tumors: epithelial (60%), germ cell (30%), and sex-cord stromal (8%) [12]. The ovarian epithelial tumors make up about 50% of all ovarian tumors, with about 40% benign and 86% malignant [9].

Benign serous tumors, which include cystadenomas, adenofibromas, and surface papillomas, account for about 25% of all benign ovarian tumors and 58% of all serous ovarian tumors [7,9].

Ovarian serous tumors are bilateral in 10% of patients. Of all serous tumors, about 70% are benign, 5%-10% have borderline malignant potential, and 20%-25% are frankly malignant. They tend to be multilocular and can include papillary projections but unilocular serous cystadenomas are not uncommon [6].

A review of racial distribution revealed that malignant epithelial cystadenomas were highest among American Indian women, followed by white, Vietnamese, Hispanic, and Hawaiian women. The incidence is lowest among Korean and Chinese women. However, no data for ovarian cysts could be obtained [13].

Clinical presentation

These masses can have a wide range of presentations that a clinician needs to be aware of. They can be entirely asymptomatic [14], or patients can present with a myriad of gastrointestinal manifestations, including nausea, abdominal pain, fullness or discomfort, or abdominal swelling and distension to even massive ascites [4,8,10,15-16]. In some cases, it can even masquerade as pregnancy in a patient of childbearing age [4]. In the elder patient, it can present with low backache, difficulty walking, anorexia, and generalized weakness [8,9,16]. The large tumor mass can also cause tachypnea and dyspnea, especially on recumbency [15].

Differential diagnoses

The differential diagnoses of giant ovarian cysts are extensive and can include benign and malignant gynecologic and non-gynecologic diagnoses [17] ranging from a distended bladder, hydronephrosis, ascites, accentuated obesity, pregnancy, fibroids, and intra-abdominal and adnexal masses.

Diagnosis

After the initial clinical evaluation, imaging modalities, such as transvaginal and transabdominal ultrasonography, computed tomography (CT), and MRI of the abdomen and pelvis are used to corroborate the diagnosis [4-5,7-10,12,15]. Clinicians can utilize the Risk of Ovarian Malignancy algorithm to determine the malignant potential of the mass and plan the management accordingly. The frozen sections and, subsequently, the histopathological evaluation will give the confirmatory diagnosis. Additional testing, such as Papanicolaou smear and endometrial sampling, would be needed.

Tumor markers

To detect the potential malignancy associated with ovarian tumors, tumor markers can be used preoperatively. CA-125, also known as mucin16, is a widely used marker and is useful in distinguishing malignant from benign pelvic masses. If a postmenopausal patient with an adnexal mass has serum CA-125 levels above 200 U/mL, the positive predictive value is of it being malignant is 96% [18]. In premenopausal patients, however, the specificity of the test is low, as the levels tend to be elevated in benign conditions as well [18]. It has been found to help in the early diagnosis of epithelial ovarian cancer with a sensitivity of 50% for patients with stage I disease [18].

Human epididymis protein 4 (HE-4) has shown superiority over CA-125 as a biomarker, specifically with its higher sensitivity to distinguish benign diseases from malignancies [19].

Management

The mainstay of treatment is the removal of the mass. This requires proper planning of the modality of approach, which can include en bloc removal versus slow percutaneous drainage. The approach depends on the size and skills of the operating surgeon while keeping in mind the risks of the development of potential surgical complications.

## Conclusions

Giant ovarian cysts are rare findings in current clinical practice, as advancement in imaging modalities and routine physical examinations have enabled clinicians to diagnose them while they are at a relatively smaller size. Our patient had predominantly vague gastrointestinal symptoms, completely unrelated to the actual diagnosis. This case highlights the importance of considering giant ovarian cysts as a differential diagnosis of vague abdominal symptoms, as these patients can have varied presentations.
